# Renal Interstitial Arteriosclerotic Lesions in Lupus Nephritis Patients: A Cohort Study from China

**DOI:** 10.1371/journal.pone.0141547

**Published:** 2015-11-06

**Authors:** Jing Huang, Sha-sha Han, Dan-dan Qin, Li-hua Wu, Yan Song, Feng Yu, Su-xia Wang, Gang Liu, Ming-hui Zhao

**Affiliations:** 1 Renal Division, Department of Medicine, Peking University First Hospital, Beijing, 100034 PR China; 2 Institute of Nephrology, Peking University, Beijing, 100034, PR China; 3 Key Laboratory of Renal Disease, Ministry of Health of China, Beijing, 100034, PR China; 4 Key Laboratory of CKD Prevention and Treatment, Ministry of Education of China, Beijing, 100034, PR China; 5 Department of Nephrology, The 2nd Affiliated Hospital, Harbin Medical University, Harbin, Heilongjiang, 150000, PR China; 6 Department of Nephrology, General Hospital of Ningxia Medical University, Yinchuan, Ningxia, 750004, PR China; 7 Department of Nephrology, the First Affiliated Hospital of Chinese PLA General Hospital, 51 Fucheng Road, Beijing, 100048, PR China; 8 Peking-Tsinghua Center for Life Sciences, Beijing, PR China; University of Utah School of Medicine, UNITED STATES

## Abstract

**Objective:**

The aim of this study was to evaluate renal arteriosclerotic lesions in patients with lupus nephritis and investigate their associations with clinical and pathological characteristics, especially cardio-vascular features.

**Design:**

A retrospective cohort study.

**Participants:**

Seventy-nine patients with renal biopsy-proven lupus nephritis, diagnosed between January 2000 and June 2008 from Peking University First Hospital.

**Results:**

In clinico-pathological data, patients with arteriosclerosis had higher ratio of hypertension and more severe renal injury indices compared with patients with no renal vascular lesions. More importantly, patients with renal arteriosclerosis had worse cardiac structure and function under transthoracic echocardiographic examination. Patients with renal arteriosclerosis tend to have higher ratios of combined endpoints compared with those of no renal vascular lesions, although the difference didn’t reach statistical meanings (*P* = 0.104).

**Conclusion:**

Renal arteriosclerotic lesion was common and associated with vascular immune complex deposits in lupus nephritis. It might have a certain degree of association with poor outcomes and cardiovascular events, which needs further explorations.

## Introduction

Systemic lupus erythematosus (SLE) is the most clinically and serologically diverse autoimmune disease, which involves multiple organs. Vascular lesion is one of the most common integral parts of the spectrum of SLE. The patients with SLE always experienced excess morbidity and mortality from cardiovascular events compared to healthy matched controls[1–4], and about 6.75% of their deaths were due to cardiovascular diseases[5]. On the other hand, the total incidence of renal involvement among patients with SLE is about 40% with variation depending on geographic area[6–7]. Renal vasculopathy might act as an “extreme” form of vascular lesions in circulation of whole SLE. Importantly, in addition to glomerulonephritis, renal vascular lesions, with various types, should be poured more attention to because their presence can adversely affect the long term renal outcomes[8–9]. Among the different renal microangiopathies, the renal interstitial arteriosclerosis is not rare, although there is still a lack of detailed descriptions in the literatures.

In this study, we evaluated renal interstitial arteriosclerosis on the basis of the 2003 International Society of Nephrology/Renal Pathology Society (ISN/RPS) classification system in a cohort of Chinese lupus nephritis patients, and further investigate its associations with clinical characteristics, especially cardiovascular features.

## Materials and Methods

Informed consent was obtained for renal biopsy from each patient. The research was in compliance with the Declaration of Helsinki. The design of this work was approved by the Medical Ethics Committee of Peking University First Hospital and informed written consent was obtained from every participant.

### Patients

Seventy-nine patients with renal biopsy-proven lupus nephritis, diagnosed between January 2000 and June 2008 from Peking University First Hospital, were enrolled. They were selected from our lupus nephritis cohort, who were performed by the transthoracic echocardiographic examination during hospitalization, and were evaluated for all types of the renal vascular lesions, including vascular immune complex deposits, arteriosclerosis, thrombotic microangiopathy, noninflammatory necrotizing vasculopathy and true renal vasculitis through renal pathological observations. Complete clinical and renal histopathological data, especially cardiovascular features and long-term outcomes, of the 79 patients were reviewed and re-classified according to the ISN/RPS 2003 classification by two experienced pathologists[10]. All the patients fulfilled the 1997 American College of Rheumatology revised criteria for SLE[11].

### Renal histopathology

The renal biopsy specimens were examined by direct immunofluorescence, light microscopy, immunohistochemistry and electron microscopy techniques.

### Direct immunofluorescence examination

Fresh frozen tissue sections were stained immediately after renal biopsy with fluorescein isothiocyanate (FITC)-labelled rabbit anti-human IgG, IgA, IgM, C3c, C1q and fibrin antibodies (DAKO A/S, Copenhagen, Denmark). Results were graded from 0 to 4 according to the intensity of fluorescence.

The vascular immune complex deposits, containing IgG, IgA, or IgM and C1q and C3, should be specially noticed.

#### Light microscopy examination

Renal biopsy specimens were fixed in 4% buffered formaldehyde for light microscopy. Consecutively the serial 3-μm sections were used for histological staining. Stains employed included haematoxylin and eosin (H&E), periodic acid-Schiff, silver methenamine (Meth) and Masson’s trichrome.

Pathological parameters such as activity indices and chronicity indices were approached by renal pathologists using a previously reported system involving semi-quantitative scoring of specific histopathological features[12,13].

**Fig 1:**
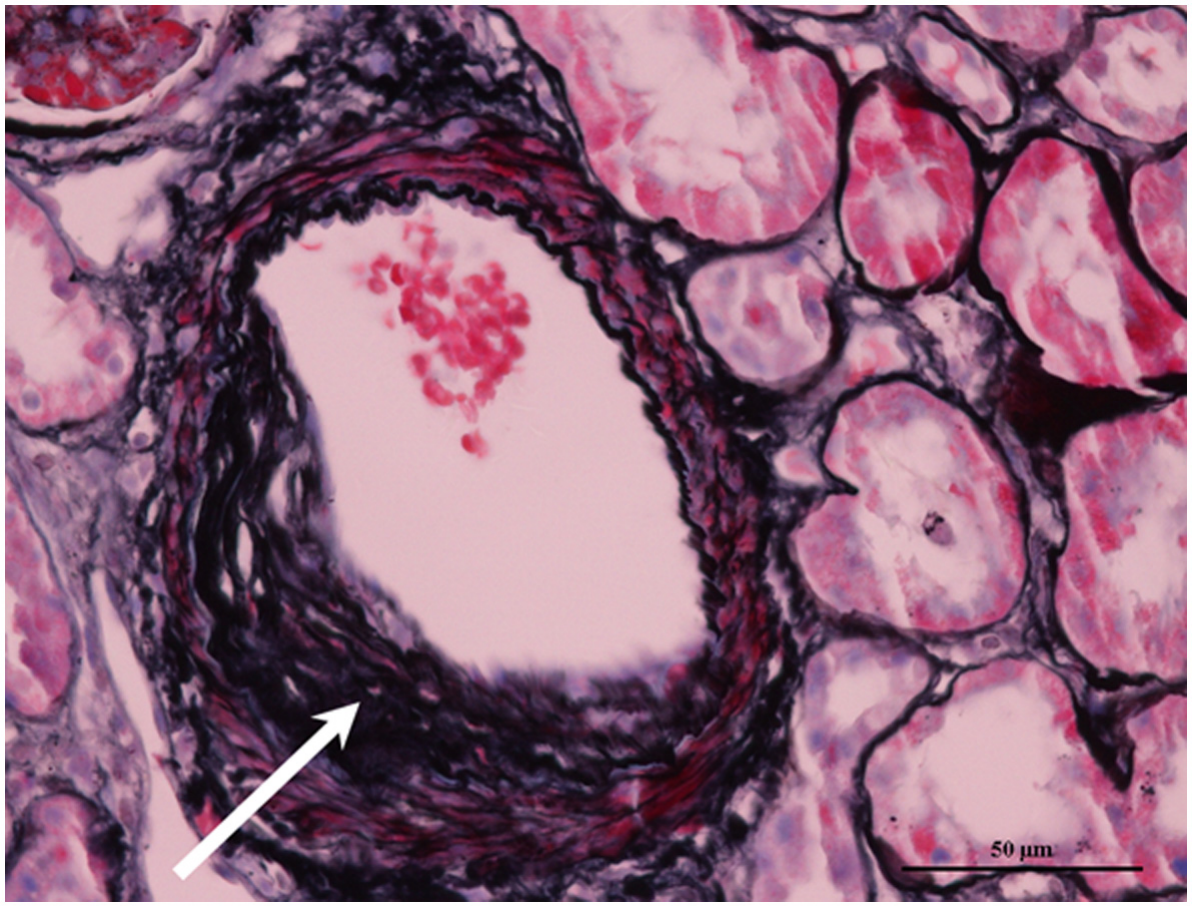
Renal interstitial vascular arteriosclerosis in a patient with lupus nephritis: Fibrous thickening of the intima of an arteriole without necrosis, proliferation, or thrombosis is seen. (arrow) (Periodic acid silver methenamine and Masson’s trichrome, original magnification ×400).

Renal interstitial arteriosclerosis was defined as fibrous thickening of the intima without necrosis, proliferation, or thrombosis (Fig 1)[14]. The semi-quantitative scores of the renal vascular arteriosclerosis was used and interlobular arterial lesions were scored based on the most severe lesions[15]. Intimal thickening was scored by comparing the thickness of the intima to that of the media in the same segment of vessel. The meaning of the lesions’ score was as follow: 0 = no intimal thickening; 1 = intimal thickened and < media thickness; 2 = intimal thickened and > media thickness.

#### Immunohistochemistry examination

To study the co-localization of arteriosclerosis and immune complex deposits in renal vessels, immunohistochemistry staining was performed using rabbit anti-human IgG and C1q antibodies (DAKO A/S, Copenhagen, Denmark) on formalin-fixed paraffin-embedded tissue (4-μm thick)[16] In briefly, optimal antibody dilutions were pre-determined, respectively. Sections were de-paraffinized and rehydrated through a series of xylene and graded alcohols. The sections were then treated with 0.4% pepsin (Zhongshan Golden Bridge Biotechnology, Beijing, China) for 40 min. Digestion was terminated by repeated washings in 0.01 mol/L phosphate buffered saline (PBS), pH 7.4. Afterwards Sections were immersed into freshly prepared 3% hydrogen peroxide in methanol solution for 10 min at room temperature to quench endogenous peroxidase activity. And subsequently to block non-specific staining, sections were incubated with 3% BSA in PBS at room temperature for 30 min. The primary antibodies against IgG (dilution 1:5000 in PBS) and C1q (dilution 1:50 in PBS) were added on each section directly. Antibodies were incubated overnight at 4°C. Horse-radish peroxidase (HRP)-labeled goat anti-rabbit IgG (Zhongshan Golden Bridge Biotechnology, Beijing, China, and dilution 1:60) were used as secondary antibodies at 37°C for 30 min respectively. Finally, the sections were counterstained with Hematein Eosin, dehydrated and cleared in alcohols and xylene, and coverslipped with permount. After all these, the sections were examined by light microscopy. Sections of renal tissues from known patients with lupus nephritis were used as positive controls. Negative controls were performed by omitting or replacing the primary antibodies.

#### Electron microscopy examination

For electron microscopy, renal biopsy specimens were fixed in 2.5% glutaraldehyde in phosphate buffer and embedded in Epon 812 resin. Ultra-thin sections were stained with uranyl acetate and lead citrate, and then examined by transmission electron microscope (JEM-1230; JEOL, Tokyo, Japan).

### Clinical and laboratory evaluations

The following clinical manifestations of lupus nephritis were analyzed: gender, age, hypertension, fever (non-infection), malar rash, photosensitivity, oral ulcer, alopecia, arthritis, serositis, neurologic disorder, anemia, leucocytopenia, thrombocytopenia, hematuria, leucocyturia (non-infection), and nephrotic syndrome. The criteria for system involvement was consistent with the 1997 American College of Rheumatology revised criteria for SLE[15]. The clinical disease activity was assessed by the Systemic Lupus Erythematosus Disease Activity Index (SLEDAI)[17,18].

#### The risk assessment of cardiovascular diseases

The relationship between renal arteriosclerotic lesions and the risk of cardiovascular diseases (CVD) in patients with lupus nephritis was estimated according to the Framingham risk score system [19]. The Framingham risk score system was on the basis of Framingham Heart Study (National Heart, Lung, and Blood Institute in Bethesda, MD, USA). It was a gender-specific scoring system used to predict an individual’s ischemic CVD risk in the next 10 years. Among several risk prediction models, the ischemic CVD outcome (i.e., coronary death, myocardial infarction, coronary insufficiency, angina, ischemic stroke, hemorrhagic stroke, transient ischemic attack, peripheral artery disease, and heart failure) was analyzed in this study. The risk score was calculated based on categorical values of age, sex, total cholesterol (TC), high density lipoprotein cholesterol (HDL-C), systolic blood pressure (SBP) and cigarette smoking.

#### Ultrasonic evaluation of heart and pulmonary artery

To evaluate the anatomic structure and functional state of the heart and pulmonary artery, the transthoracic echocardiographic examination was performed in all the 79 patients using GE scanner (VIVIDE9), according to the standard protocol described in the American Echocardiographic Guideline[20]. All echocardiographic examinations were performed by the same experienced echocardiographer, who was blinded to clinical data of the patients. The measured parameters with the M-mode technique included left atrium diameter, left ventricular end-diastolic diameter, interven-tricular septum and left ventricular posterior wall thickness and calcification of valves. The measurement of left ventricular ejection fraction was undertaken by the Simpsons biplane method. Early and late mitral peak inflow velocity and E wave deceleration time were assessed in apical four-chamber views using pulsed wave Doppler beam at the level of mitral valve leaflet tips. The pulmonary arterial systolic pressure was calculated by adding the estimated right atrial pressure to pulmonary arterial systolic pressure. All the ultrasound findings were verified by a sonographer who was blinded to all of the cases.

### Laboratory assessment

The levels of blood lipids, including triglyceride (TG) (BIOSINO BIO-TECHNOLOGY&SCIENCE INC, Beijing, China; normal range: 0.56–1.7mmol/L),total cholesterol (TCHO) (BIOSINO BIO-TECHNOLOGY&SCIENCE INC, Beijing, China; normal range: 3.4–5.2mmol/L), high-density lipoprotein cholesterol (HDL-C) (SEKISUI MEDICAL TECHNOLOGY (CHINA) LTD, Beijing, China; normal range: 0.9–1.4mmol/L) and low-density lipoprotein cholesterol (LDL-C) (SEKISUI MEDICAL TECHNOLOGY (CHINA) LTD, Beijing, China; normal range: 2.1–3.1mmol/L), for all the patients were detected before the day of renal biopsy.

Serum antinuclear antibodies (ANA) were detected using indirect immunofluorescence assay (EUROIMMUN, Lübeck, Germany) and anti-double-stranded DNA antibodies were detected using Crithidia luciliae indirect immunofluorescence test (EUROIMMUN, Lübeck, Germany). Anti-extractable nuclear antigen (ENA) antibodies, including anti-Sm, anti-SSA, anti-SSB and anti-RNP antibody, were detected using immunodotting assay (EUROIMMUN, Lübeck, Germany). Anti-cardiolipin antibodies were detected using ELISA (enzyme-linked immunosorbent assay) (EUROIMMUN). Serum C3 was determined using rate nephelometry assay (Beckman-Coulter, IMMAGE, USA, normal range>0.85g/L).

### Treatment response and follow-up

The response to therapy includes complete remission, partial remission and treatment failure detailed in previous works[21–23]. A relapse was defined as: 1) nephritic relapse: a recent increase of serum creatinine by >50% with active urinary sediments; 2) proteinuric relapse: development of either a nephrotic syndrome (proteinuria >3.5g/day and serum albumin <30g/L) or proteinuria >1.5g/day without other causes, in previously non-proteinuric patients[24,25].

The patients were followed up in outpatient lupus clinics. The combined endpoints included death and major cardiovascular events, which consisted of cardiovascular events (heart failure, coronary artery disease, stroke or cerebral hemorrhage) and renal events (end-stage renal disease or doubling of serum creatinine) [26].

### Statistical analysis

Statistical software SPSS 13.0 (SPSS, Chicago, IL, USA) was used for statistical analysis. Quantitative data were expressed as mean ± s.d. (for data that were normally distributed), and median with range (minimum, maximum) (for data that were not normally distributed). Differences of quantitative parameters data were tested with the independent-samples T tests and one-way ANOVA tests (for data that were normally distributed), or with the Kruskal–Wallis test and the Mann–Whitney U-test (for data that were not normally distributed). Kaplan–Meier curves were used to analyze patients’ prognosis. Univariate survival analysis was carried out using the log-rank test. Multivariate analysis of combined outcomes was performed using the Cox regression model. Statistical significance was considered as *P*<0.05.

## Results

### General clinical data of patients with lupus nephritis

The clinical and pathological data of all the 79 patients were summarized in Table 1. The average age of the patients at the time of renal biopsy was 33.5 ± 10.8 years (range 12–72 years). The male-to-female ratio was 1:6.9.

**Table 1:** Clinical and pathological data of lupus nephritis patients.

Clinical evaluation		Laboratory assessment		Renal histopathology indices	
Gender (male/female)	10/69	Leukocytopenia No. (%)	32(40.5)	Activity indices (AI) score (median, range)	7.8 ±4.4
Age (mean ± SD) (years)	33.5±10.8	Thrombocytopenia No. (%)	29 (37.2)	Endocapillary hypercellualrity (median, range)	3 (1, 3)
Follow-up time (median, range)(months)	36.0(12.0, 72.0)	Hematuria No. (%)	63(79.7)	Cellular crescents (median, range)	0 (0, 2)
Fever (non-infectious) No. (%)	23(29.1)	Leukocyturia (non-infection) No. (%)	32 (40.5)	Karyorrhexis/fibrinoid necrosis(median, range)	0 (0, 2)
Malar rash No. (%)	36 (45.6)	Hemoglobin (mean ± s.d.) (g/l)	98.83±26.88	Subendothelial hyaline deposits (median, range)	1 (0, 2)
Photosensitivity No. (%	15(19.2)	Urine protein (median, range) (g/24hours)	4.86 (2.94,7.70)	Interstitial inflammatory cellinfiltration(median,range)	1 (1, 1)
Oral ulcer No. (%)	20(25.3)	Serum creatinine (median, range) (μmol/l)	84 (69,139)	Glomerular leukocyte infiltration (median, range)	1 (0,1)
Alopecia No. (%)	22(27.8)	Creatinine clearance rate (median,range)(ml/min)	73(54,)	Chronicity indices (CI) score (median, range)	2.7 ±1.9
Arthralgia No. (%)	35(44.9)	Anti-cardiolipin antibody (+) No. (%)	7(11.5)	Glomerular sclerosis (median, range)	0 (0, 1)
Serositis No. (%)	13(16.7)	Anti-nuclear antibody (+) No. (%)	77(97.5)	Fibrous crescents (median, range)	0 (0, 0)
Neurologic disorder No. (%)	5 (6.5)	Anti-double stranded DNA antibody (+) No. (%)	45(57)	Tubular atrophy (median, range)	1 (1,1)
Anemia No. (%)	49(62.8)	Anti-SSA antibody (+) No. (%)	35(44.3)	Interstitial fibrosis (median, range)	1 (1 1)
Hypertension No. (%)	31(39.2)	Anti-SSB antibody (+) No. (%)	10(12.7)		
Acute renal failure No. (%)	18 (22.8)	Anti-Smith antibody(Sm) (+) No. (%)	19 (24.1)		
Nephrotic syndrome No. (%)	49(72.16)	Anti-ribonucleoprotein(RNP) antibody (+)No. (%)	23(29.1)		
SLEDAI (mean ± s.d.)	16.85±5.66				
Framingham risk score system(mean and range)	7(3,12)				

Note: SLEDAI: Systemic Lupus Erythematosus Disease Activity Index

According to the 2003 International Society of Nephrology/Renal Pathology Society classification system for lupus nephritis[10], 2 patients were classified as class II (2.5%), 16 as class III (20.2%, including 8 as class V+III), 47 as class IV (59.5%, 9 as class IV-segmental (IV-S) and 38 as class IV-global (IV-G), including 9 as class IV+V), and 14 as class V (17.7%). None was in class I or class VI in this study.

All of the patients received oral prednisone therapy. The majority of patients completed treatment with oral cyclophosphamide or monthly intravenous Cyclophosphamide (600–800mg/month) (60/79). The other patients received mycophenolate mofetil (6/79), leflunomide (10/79) and azathioprine (3/79). Most of the patients achieved clinical remission, including 41 with complete remission and 23 with partial remission. Fifteen patients presented with treatment failure.

For the management of blood pressure, 31 out of the 79 patients with hypertension took oral antihypertensive drugs, including 28 patients with calcium antagonists, 15 with Beta blockers, 6 with angiotensin receptor blocker and 6 with angiotension-converting enzyme inhibitor. Among them, 9 patients used single antihypertensive agent and 22 patients with at least 2 types of drugs. In totally, 25 of the 31 patients kept in good control of blood pressure and 6 were in poor management.

The average follow-up time was 57.0 months (range 6–360 months). With regard to long-term prognosis, 15 patients (19%) reached combined endpoints, including 1 (1.3%) patient died, 5 (6.3%) with coronary artery disease, 3 (3.8%) with heart failure and 6 (7.6%) with doubling of serum creatinine.

### The distribution of renal interstitial arteriosclerosis in lupus nephritis

**Fig 2:**
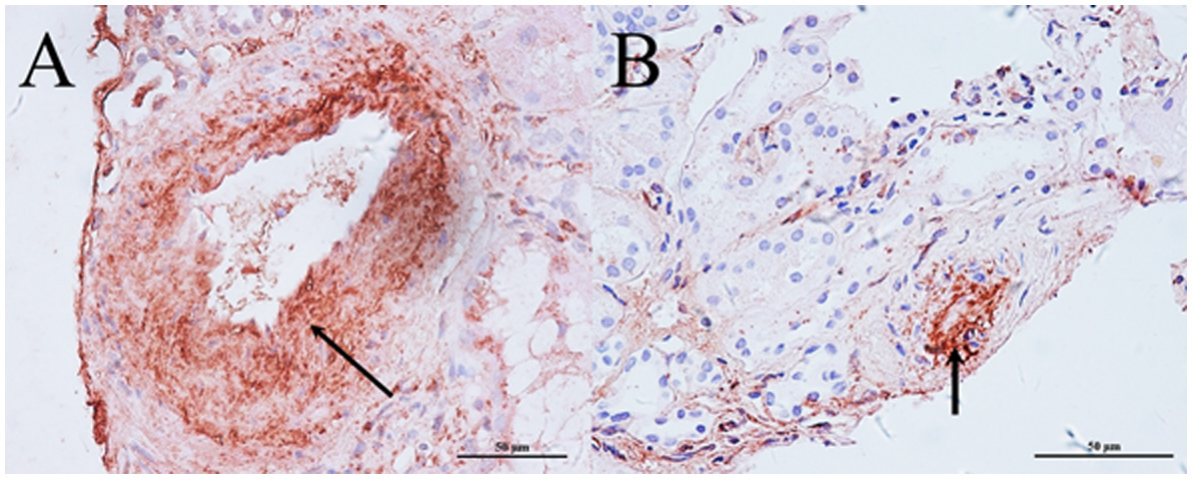
Co-localization of arteriosclerosis and immune complex deposits in renal vessels. (A). IgG was present in the arteriosclerotic lesion of extra-glomerular arteriole (arrow); (B). C1q was present in the arteriosclerotic lesion of extra-glomerular arteriole (arrow). (Immunohistochemistry, original magnification ×400).

Among the 79 patients with lupus nephritis, 50 patients were with arteriosclerosis lesions and 29 patients were without any vascular changes including vascular immune complex deposits, arteriosclerosis, thrombotic microangiopathy, noninflammatory necrotizing vasculopathy and true renal vasculitis. For patients with arteriosclerosis, 27 patients were scored as 1, and the other 23 patients were scored as 2. Especially, of the 50 patients with arteriosclerosis, 34 also combined with vascular immune complex deposits, including 20 with IgG, 7 with IgA, 18 with IgM, 17 with C3c, 32 with C1q, and 22 with C4d deposits. As most lupus nephritis patients in the study suffered both arteriosclerosis and vascular IgG or C1q deposits, we further detect the co-localization of them in renal vessels. The immuno-histochemistry examination showed that IgG or C1q was present in the wall of extra-glomerular arteries and arterioles which had arteriosclerotic lesion (Fig 2).

### Comparisons of clinical and pathological data in patients with arteriosclerosis and without renal vascular lesions

We further compared the clinical and pathological characteristics of patients with arteriosclerosis to those without renal vascular lesions.

For patients in the two groups, there was no significant difference in most clinical features at the time of renal biopsy (Table 2). However, we found that patients with arteriosclerosis presented with higher propotion of hypertension compared to patients with no renal vascular lesion group (48% (24/50) vs. 24.14% (7/29), *P* = 0.036).

**Table 2:** Comparisons of clinical and laboratory data in patients with arteriosclerosis and without renal vascular lesions.

	AS	NRVL	*P*-value
Number of patients	50	29	
Gender (male/female)	6/44	4/25	1.000
Age (median and range) (years)	32(17, 57)	33(15, 64)	0.835
Number with leukocyturia (non-infection) (%)	23 (46.0)	17 (58.6)	0.352
Number with hematuria (%)	43 (86.0)	20 (69.0)	0.086
Number with acute renal failure (%)	14 (28.0)	4 (13.8)	0.174
Number with hypertension (%)	24 (48%)	7 (24.14%)	0.036
Hemoglobin (mean ± s.d.) (g/L)	93.46 ± 29.22	110.57 ± 21.47	0.007
Platelets (10^9^/L)	147.19 ± 101.45	196.04 ± 119.70	0.095
Urine protein (mean ± s.d.) (g/24 h)	5.7 ± 3.94	5.27 ± 3.46	0.722
Albumin(g/L)	23.54 ± 7.52	24.72 ± 7.00	0.342
Serum creatinine (mean ± s.d.) (μmol/L)	145.17 ± 131.52	92.03 ± 55.64	0.009
Number with positive ANA (%)	49 (98.0)	28 (96.6)	1.000
Number with positive anti-ds-DNA antibodies (%)	32 (64.0)	13 (44.8)	0.107
Number with positive anti-Sm antibodies (%)	7(14.0)	12(41.4)	0.008
Number with positive anti-cardiolipin antibodies (%)	6(7.9)	4(16.7)	0.415
TG (mmol/L)	2.90 ±1.86	2.86 ± 1.57	0.755
TCHO (mmol/L)	6.34 ± 2.33	6.66 ± 2.32	0.550
HDL-C (mmol/L)	1.18 ± 0.49	1.17 ± 0.61	0.618
LDL-C (mmol/L)	3.59 ± 1.53	3.72 ± 1.26	0.602
Framingham risk score system(median; inter-quartile range)	7(2,12)	8.5(3,11.25)	0.971
SLEDAI	16.86±1.13	16.55±5.32	0.947

Note: AS: arteriosclerosis; NRVL: no renal vascular lesion; TCHO: total cholesterol; TG: triglyceride; HDL-C: high-density lipoprotein cholesterol; LDL-C: low-density lipoprotein cholesterol; SLEDAI: Systemic Lupus Erythematosus Disease Activity Index.

The laboratory was suggestive of that patients with arteriosclerosis presented with lower level of hemoglobin, higher level of serum creatinine and lower ratio of anti-Sm antibodies positive, compared with the other group (93.46 ± 29.22 g/L vs. 110.57 ± 21.47 g/L, *P* = 0.007; 145.17 ± 131.52 μmol/L vs. 92.03 ± 55.64 μmol/L, *P* = 0.009; 14% vs. 41.4%, *P* = 0.008, respectively). However, there were no significant differences in the levels of serum lipids between the two groups. Furthermore, for the risk assessment of CVD, there were also no significant differences of the Framingham risk scores between them (Table 2). Nevertheless, as to the echo-cardiographic parameters, patients with arteriosclerosis presented with more severe indices, including larger left atrium diameter, left ventricular end-diastolic diameter, interventricular septum and left ventricular posterior wall (*P* = 0.010; *P* = 0.008; *P* = 0.047; *P* = 0.003, respectively), compared with the other group (Table 3).

**Table 3:** Comparisons of echo-cardiographic parameters in patients with arteriosclerosis and without renal vascular lesions.

	AS	NRVL	*P*-value
Number of patients	50	29	
LA(cm)	3.46 ± 0.48	3.19 ± 0.42	0.010
Aortic diameter (cm)	2.64 ± 0.38	2.51 ± 0.35	0.201
LVEDD(cm)	5.02 ± 0.55	4.71 ± 0.43	0.008
IVS(cm)	0.98 ± 0.18	0.90 ± 0.10	0.047
LVPW(cm)	0.99 ± 0.15	0.89 ± 0.09	0.003
LVEF (%)	64.70 ± 12.26	69.76 ± 0.25	0.127
E peak (cm/s)	93.60 ± 22.66	88.28 ± 15.81	0.281
A peak (cm/s)	70.45 ± 16.30	72.14 ± 12.74	0.346
EDT (ms)	183.30 ±34.23	187.4 ± 62.84	0.623
PASP(mmHg)	29.07 ± 6.52	27.42 ± 4.08	0.356
Aortic valve calcification (%)	7 (14.3)	4 (13.8)	1.000
Mitral valve calcification (%)	15 (30.0)	5 (17.2)	0.171
Aortic regurgitation (%)	15 (30.0)	10 (34.5)	0.803
Mitral regurgitation (%)	31 (62.0)	18 (62.1)	1.000

Note: AS: arteriosclerosis; NRVL: no renal vascular lesion; LA: left atrium diameter; LVEDD: left ventricular end-diastolic diameter; IVS: interven-tricular septum; LVPW: left ventricular posterior wall; LVEF: left ventricular ejection fraction; EDT: E wave deceleration time; PASP: pulmonary arterial systolic pressure.

In renal pathological data, patients with arteriosclerosis had higher scores of cellular crescents, interstitial inflammatory cell infiltration and fibrous crescents (*P* = 0.026; *P* = 0.012; *P* = 0.020, respectively) (Table 4). The scores of renal vascular arteriosclerosis lesions were mildly positively correlated with the scores of total activity indices(r = 0.288, *P* = 0.010), cellular crescents (r = 0.331, *P* = 0.003), and interstitial inflammatory cell infiltration (r = 0.270, *P* = 0.016). Similarly, the scores of renal vascular arteriosclerosis lesions were also positively correlated with the thickness of left atrium diameter (r = 0.510, *P* = 0.026), interven-tricular septum (r = 0.299, *P* = 0.007), and left ventricular posterior wall (r = 0.397, *P*<0.001).

**Table 4:** Comparisons of pathological data in patients with arteriosclerosis and without renal vascular lesions.

	AS	NRVL	*P*-value
Number of patients	50	29	
Arteriosclerosis score (mean ± s.d.)	1.46± 0.53	0	<0.001
Endocapillary hypercellularity(median; inter-quartile range)	3;1.75–3	2;1–3	0.295
Cellular crescents (median; inter-quartile range)	1;0–4	0;0–2	0.026
Karyorrhexis/fibrinoid necrosis (median; inter-quartile range)	0.5;0–2	0;0–2	0.376
Subendothelial hyaline deposits (median; inter-quartile range)	1;0–2	1;0–1	0.773
Interstitial inflammatory cellinfiltration (median; inter-quartile range)	1;1–2	1;1–1	0.012
Glomerular leukocyte infiltration (median; inter-quartile range)	1;1–1	1;0–1	0.085
Total Activity indices (AIs) score(median; inter-quartile range)	8.5;4–12	6;4–9	0.071
Glomerular sclerosis (median; inter-quartile range)	0;0–1	0;0–1	0.297
Fibrous crescents (median; inter-quartile range)	0;0–0.25	0;0–0	0.020
Tubular atrophy (median; inter-quartile range)	1;1–1	1;1–1	0.853
Interstitial fibrosis (median; inter-quartile range)	1;1–1	1;1–1	0.823
Total Chronicity indices (CIs)score (median; inter-quartile range)	3;2–4	2;2–3	0.198

Note: AS: arteriosclerosis; NRVL: no renal vascular lesion.

### Comparisons of treatment and outcomes between patients with arteriosclerosis and without renal vascular lesions

There was no significant difference in the treatment algorithm between the two groups (Table 5). The ratio of the blood pressure control was not significantly different between patients with arteriosclerosis and with no renal vascular lesion (79.2% (19/24) vs. 85.7% (6/7), *P* = 0.692), and the arteriosclerosis group need more antihypertensive drugs than that in no renal vascular lesion group (2.5 (2, 4) vs. 1 (1, 2), *P* = 0.043). Immunologic treatment response was similar between the two groups. During a similar follow-up time (average for nearly 5 years), the relapse rates between the groups were not significantly different.

**Table 5:** Comparisons of treatment between patients with atherosclerosis and without renal vascular lesions.

	AS	NRVL	P-value
Number of patients	50	29	
Treatment				
P (Number of patients (%))	50 (100%)	29 (100%)	
CYC (Number of patients(%))	38 (76.0%)	22 (75.9%)	0.989
AZA (Number of patients(%))	2 (4.0%)	1 (3.4%)	1.000
MMF(Number of patients(%))	4 (8.0%)	2 (6.9%)	0.858
LEF (Number of patients (%))	6 (12.0%)	4 (13.8%)	0.817
Treatment response				
CR (Number of patients (%))	25 (50.0%)	16 (55.2%)	0.657
PR (Number of patients (%))	15 (30.0%)	8 (27.6%)	0.820
TF (Number of patients (%))	10 (20.0%)	5 (17.2%)	0.763
Control of hypertension (%)	19/24 (79.2%)	6/7 (85.7%)	0.692
Duration of follow-up(mean and range) (months)	61.8 (6, 214)	54.1 (6,360)	0.222
Relapse rate (%)	3/40 (7.5%)	3/24 (12.5%)	0.664

Note: AS: arteriosclerosis; NRVL: no renal vascular lesion; P: oral prednisone; CYC: cyclophosphamide; AZA: azathioprine; MMF: mycophenolate mofetil; LEF: leflunomide; CR: complete remission; PR: partial remission; TF: treatment failure.

**Fig 3:**
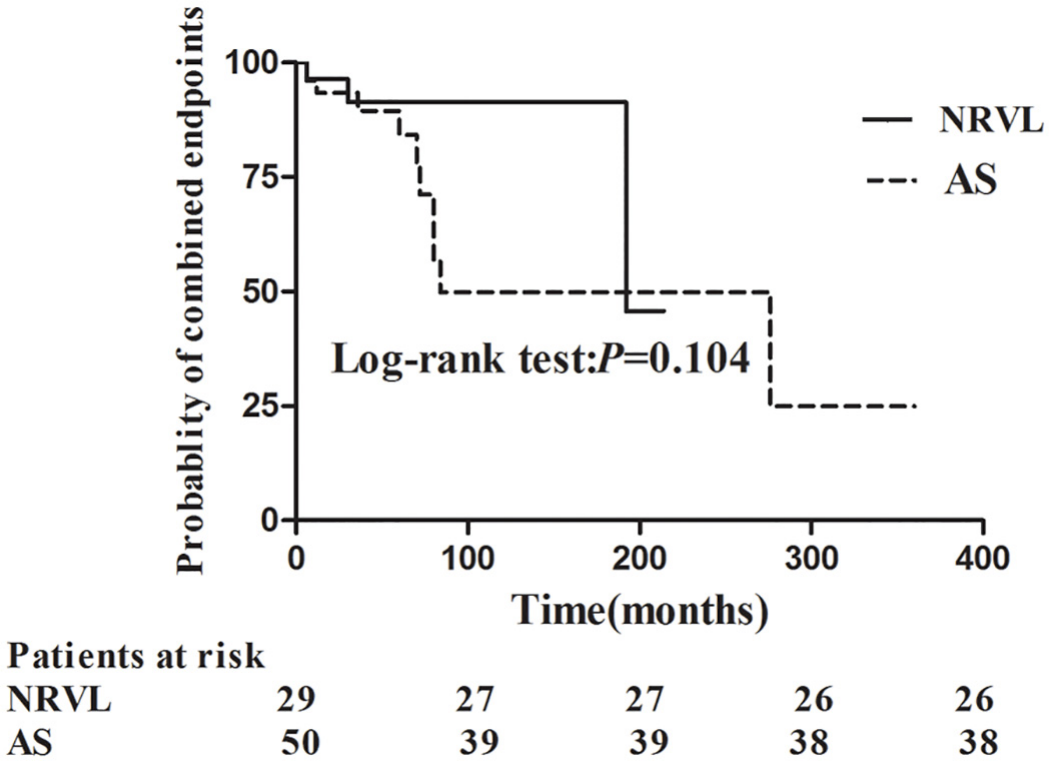
Comparison of ratios of combined endpoints between patients with arteriosclerosis and without renal vascular lesions. (AS: arteriosclerosis; NRVL: no renal vascular lesion).

For the analysis of occurrence of combined endpoints during follow-up, 12 patients (24%) reached the endpoints in arteriosclerosis group, including 1 (2%) patient died, 3(6%) with coronary artery disease, 3 (6%) with heart failure and 5 (10%) with doubling of serum creatinine; in no renal vascular lesion group, 3 patients (10.3%) reached combined endpoints, including 2 (6.9%) with coronary artery disease and 1 (3.4%) with doubling of serum creatinine. Patients with arteriosclerosis tend to have worse prognosis although the ratio was not significantly different between the two groups (*P* = 0.104) (Fig 3).

For univariate survival analysis of combined endpoints in the lupus nephritis patients, we found that the following cardio-vascular parameter, including the left atrium diameter, the left ventricular end-diastolic diameter and mitral regurgitation, were risk factors for outcomes (*P* = 0.032; *P* = 0.0.019; *P* = 0.023, respectively). Renal arteriosclerosis was not a risk factor (*P* = 0.515) (Table 6). In a further multivariate Cox hazard analysis of the above indices, the thickness of left ventricular end-diastolic diameter and serum creatinine level, were identified as independent risk factors for combined endpoints by adjustment using a stepwise model (hazard ratio: 13.564, 95% confidence intervals: 1.497–122.889, *P* = 0.020; hazard ratio:1.006, 95% confidence intervals: 1.001–1.011, *P* = 0.027) (Table 7).

**Table 6:** Univariate survival analysis of combined events in patients with lupus nephritis.

	*P*-value	95% confidence intervals		Hazard ratio
		Lower	Upper	
Sex	0.315	0.521	7.540	1.983
Age	0.043	0.902	0.998	0.949
Hemoglobin	0.412	0.982	1.008	0.995
Urine protein	0.728	0.908	1.148	1.021
Serum creatinine	0.019	1.001	1.007	1.004
Anti-ds-DNA antibodies	0.066	0.926	10.407	3.104
Anti-SSB antibodies	0.134	0.719	11.917	2.928
SLEDAI	0.206	0.968	1.162	1.061
Total Activity indices (AIs) score	0.115	0.978	1.231	1.097
Total Chronicity indices (CIs)score	0.493	0.846	1.414	1.094
Renal arteriosclerosis	0.515	0.415	5.778	1.548
Acute renal failure	0.059	0.956	10.851	3.220
HDL-C	0.527	0.054	4.466	0.490
LA	0.032	1.114	11.004	3.501
LVEDD	0.019	1.274	15.277	4.411
IVS	0.073	0.810	120.778	9.890
LVPW	0.392	0.121	219.368	5.149
Mitral regurgitation	0.023	1.291	29.483	6.170
Aortic calcification	0.089	0.823	15.062	3.522

Note: SLEDAI: Systemic Lupus Erythematosus Disease Activity Index., HDL-C: high-density lipoprotein cholesterol; LA: left atrium diameter; LVEDD: left ventricular end-diastolic diameter; IVS: interven-tricular septum; LVPW: left ventricular posterior wall.

**Table 7:** Multivariate analysis of the risk factors for combined events in patients with lupus nephritis.

	*P*-value	95% confidence intervals		Hazard ratio
		Lower	Upper	
Multivariate Cox hazard analysis				
Age	0.090	0.746	1.021	0.873
Sex	0.389	0.395	10.883	2.074
Acute renal failure	0.057	0.003	1.094	0.058
Activity indices score	0.057	0.990	1.860	1.357
Chronicity indices score	0.350	0.379	1.409	0.731
Serum creatinine	0.019	1.001	1.016	1.009
LVEDD	0.073	0.817	95.421	8.829
Renal arteriosclerosis	0.057	0.000	1.120	0.019
Multivariate stepwise Cox hazard analysis				
Serum creatinine	0.027	1.001	1.011	1.006
LVEDD	0.020	1.497	122.889	13.564

Note: LVEDD: left ventricular end-diastolic diameter.

## Discussion

SLE is an autoimmune disease with protean clinical and pathologic manifestations involving almost all organs in the body. Previous studies reported that nearly 30% of patients with SLE had vascular disease, in which cardiovascular events contributed a lot to the late-stage mortality peak of SLE patients[5,26]. Epidemiological studies also revealed a precocious, accelerated arteriosclerosis in SLE[5]. As an extreme form of vascular lesions in circulation of whole SLE, several studies, including ours, indicated that renal vasculopathy could also be identified in a large part of lupus nephritis patients[14,16,20]. Renal arteriosclerosis was one of the major renal vascular lesions, although the descriptions of it in lupus nephritis patients were few and the pathogenesis remained unclear. Several studies showed that chronic inflammation became the focus of attention in both early and advanced atherogenic processes, and it also played an important role in the process of arteriosclerosis. For immune complexes in autoimmune backgrounds, either autoantibodies against self-molecules or new epitopes in circulating or in situ, they could modulate the activity of immune cells and release the inflammatory mediators, such as TNF-α, IL-1, *etc*. They had the prominent immunomodulatory properties which might influence the atherosclerotic inflammation and atherogenesis itself, and be involved in the pathogenesis of cardiovascular events in some rheumatic diseases, especially SLE[27,28].

Our study found that renal arteriosclerosis change was common, which was higher than 60%, in pathological findings of lupus nephritis. The patients with the lesions presented with more severe renal injury and higher ratio of hypertension compared with those without renal vascular lesions, although the levels of serum lipids and the Framingham risk scores were not significantly different. More interestingly, we found that higher than 1/3 of the patients with renal arteriosclerosis also combined with vascular immune complex deposits, and our immuno-histochemistry examination further identified that arteriosclerosis and IgG or C1q could co-localize in renal vessels. Our findings suggested that the immune complex and hypertension might be associated with the formation of renal arteriosclerosis in lupus nephritis. As we know, patients with lupus nephritis are characterized by the production of a variety of nephrogenic autoantibodies, which could bind to glomerular basement membrane, endothelial cells and mesangial cells, or as the form of immune complex from circulation, and deposited in the kidneys, subsequently aggravating inflammation response via complement pathways, etc, and resulting in inflammatory injury[29,30]. Increasing evidences indicated that the complement system could be activated within atherosclerotic plaques[31]. Recently, Lewis et al. found that, in a mice model combining with lupus and hyperlipidemia, accelerated arteriosclerosis of aorta was related to complement activation and reduced uptake and removal of apoptotic/necrotic debris[32].

Previous studies indicated that the incidence of myocardial infarctions was 9 times more common in patients with SLE than controls, and patients with general chronic kidney disease were more likely to die from cardiovascular disease than end stage renal disease[33,34]. However, it is still not yet clear of the relationship between renal arteriosclerosis lesions, one of the most common types of renal vascular injury, and systemic cardiovascular event in lupus nephritis. Thus, with further analysis in our study, we found that the patients with arteriosclerosis presented with worse cardiac structure and function under transthoracic echocardiographic examination. Furthermore, the scores of renal arteriosclerosis were significantly positively correlated with left atrium diameter, interven-tricular septum, and left ventricular posterior wall thickness. By a univariate survival analysis, we found that several echo-cardiographic parameters were risk factors for combined endpoints in the long follow-up time, and in a further multivariate Cox hazard analysis, left ventricular end-diastolic diameter was demonstrated as an independent risk factor for worse outcomes, although renal arteriosclerosis score was not. Likely, in a previous study of hemodialysis patients, the left ventricular end-diastolic diameter was also found as an independent predictor of cardiovascular mortality[35]. Importantly, our data also showed that lupus nephritis patients with arteriosclerosis presented with more cardiovascular events than those without any renal vascular lesion, although the difference didn’t reach statistical meanings, which might be attributed to the limited sample size from the single center.

Although there existed pathogenic complexity, some potential common mechanic pathways focus on the association between renal vessel injury and cardiovascular impairment in SLE were proposed in recent literatures. Vasculopathy in SLE, including cardiovascular and renal vascular diseases, may be of inflammatory or thrombotic origin. Atherosclerosis or ateriosclerosis might also be an inflammatory disease[36]. The dysfunction of immune system is considered to be the predominant feature of this disease, including that (1) The immune complexes deposited onto the vascular endothelium and triggered the inflammatory response. (2) Various autoantibodies directly or indirectly affected the endothelial cells and cause chronic vessel wall damage[37,38]. (3) Some cytokines, such as type I interferon or TNF-α [39], could also injury the endothelium, *etc*. Moreover, numerous lines of evidence suggested that the activation and injury of endothelial cells might play a key role in the pathogenesis of vasculopathy in SLE, as endothelial dysfunction represented a state of deviation from normal to a vasoconstrictive, procoagulant, platelet activating, and anti-fibrinolytic state[40,41]. Thus, we proposed that the association between cardiac and renal vascular injury in SLE was complex and might be mediated by an interaction between immune system and other factors. However, the precise mechanism of these diseases has not been fully elucidated, which needs further explorations.

Taken together, we proposed that the renal arteriosclerosis might predict potent cardiovascular risk in lupus nephritis, which need further investigations. In recent years, there were a growing number of studies underscoring the importance of multidisciplinary approach, including the control of blood pressure, lipids, uric acid, etc, for patients with lupus nephritis, which would improve the total prognosis[42]. Thus, the assessment of renal arteriosclerosis in biopsy might provide more evidence for the judgement of systemic treatment strategy in lupus nephritis.

However, with the limitations of this retrospective analysis, the prospective multi-center study with larger sample size was needed, especially focus on the combined evaluations of both renal injury and cardiovascular indices such as carotid plaque/carotid intima-media thickness or arterial stiffness, etc.

In conclusion, renal arteriosclerotic lesion was common and associated with vascular immune complex deposits in lupus nephritis. It might be associated with poor outcomes and cardiovascular events in some extent, which needs further explorations.
